# Using neuroscience techniques to understand and improve design cognition

**DOI:** 10.3934/Neuroscience.2020018

**Published:** 2020-08-24

**Authors:** Evangelia G. Chrysikou, John S. Gero

**Affiliations:** 1 Department of Psychology, Drexel University, 3201 Chestnut St., Philadelphia, PA 19140, USA; 2 Department of Computer Science and School of Architecture, University of North Carolina at Charlotte, 9201 University City Blvd, Charlotte, NC 28223, NC, USA

**Keywords:** cognitive neuroscience, design thinking, problem-solving and creativity, functional magnetic resonance imaging, transcranial electric stimulation

## Abstract

Cognitive neuroscience research has traditionally focused on understanding the brain mechanisms that enable cognition by means of experimental laboratory tasks. With a budding literature, there is growing interest in the application of the related methods and findings to real-world settings. In this opinion paper we explore the potential and promise of employing current cognitive neuroscience methodologies in the field of design. We review recent evidence from preliminary studies that have employed such methods toward identifying the neural bases of design thinking and discuss their impact and limitations. Further, we highlight the importance of pairing neuroscience methods with well-established behavioral paradigms during ecologically-valid, real-world design tasks. Experimental investigations that meet these requirements can generate powerful datasets of neurocognitive measures that can offer new insights into the complex cognitive and brain systems enabling design thinking. We argue that this new knowledge can lead to the development and implementation of new techniques toward cultivating and improving design thinking in design education and professional practice.

## 1. Introduction

Cognitive neuroscience is the subfield of neuroscience that focuses on the neural mechanisms enabling mental processes—from perception and memory, to higher-order thinking and problem solving. For a little over three decades, cognitive neuroscience methodologies—including structural and functional brain imaging, electroencephalography, and noninvasive brain stimulation—have allowed researchers to capture *in vivo* patterns of neural activity associated with these complex mental operations. Some of these techniques have also occasionally provided evidence for causal brain-behavior relationships that have supported interventions to alter, treat, or augment brain function in healthy and diseased populations [1]. Although the majority of research in cognitive neuroscience focuses on our understanding of brain mechanisms and circuits that give rise to cognition through traditional cognitive psychology paradigms and tasks, there is growing interest in the application of the related methods and findings for real-world, ecologically-valid, educational and professional settings. The present opinion paper focuses on the potential and promise of employing modern cognitive neuroscience methodologies in the field of design. We will first offer a brief overview of the types of neurophysiological methods that have recently been used within the fields of design cognition and neurocognition. We will then discuss some of the findings of this extant work and their and limitations and propose new directions for the meaningful pairing of cognitive neuroscience methodologies with ecologically-valid, real-world design tasks. We will highlight how the productive integration of cognitive neuroscience and design methods holds strong potential to generate powerful datasets of neurocognitive measures that can provide a unique understanding of the complex cognitive and brain systems enabling design thinking. We will conclude with a discussion of how this new knowledge can be manifested in the opportunity to develop and implement new techniques toward cultivating and improving design thinking in design education and professional practice.

## 2. Cognitive neuroscience methods in applied design settings

Designing is the cognitive act of intentionally generating new ways to change the world instead of simply repeating existing ways. It is ubiquitous—carried out by both professional designers and by anyone who executes the same cognitive acts [2]. How do designers conceive and develop new ideas? How do these ideas evolve in the process of designing? *Design thinking* pertains to the neurocognitive processes supporting the development of design concepts (e.g., for products or services) from their inception to their final description [3]. The process of designing reflects one of the most complex aspects of higher-order human cognition and it is widely considered a cornerstone of human creativity [4],[5]. Understanding design thinking has been at the center of design studies for nearly half a century, with an emphasis on protocol analysis methods adopted from cognitive psychology [6]–[8]. Although such techniques have propelled design research at the behavioral level and have significantly advanced our understanding of the characteristics of design cognition descriptively, a comprehensive investigative approach to design thinking at different levels of analysis still remains elusive. Recently, the development of the interdisciplinary field of *design neurocognition* has introduced new possibilities for understanding design thinking through the integration of traditional design research techniques such as protocol analysis, with methods from cognitive neuroscience, neurophysiology, and artificial intelligence [9],[10].

Design research involving cognitive neuroscience methods has typically employed techniques that allow for the recording of neural activity while designers think and as they generate design products. Such techniques typically entail methods that emphasize spatial resolution—namely, where in the brain a particular process may take place, whereas others emphasize temporal resolution—namely, when a particular neural process occurs relative to the task performed, with some techniques combining both approaches or emphasizing the organization of brain regions in large-scale networks [11]. Among the measures of high spatial resolution is structural and functional magnetic resonance imaging (MRI, and fMRI, respectively), with the former highlighting brain structure-behavior relationships and the latter ongoing brain activity during cognition. Measures of high temporal resolution include those capturing the electrical activity of the brain, namely electroencephalography [EEG] and its extension, event-related potentials [ERP]. Functional near-infrared spectroscopy [fNIRS] approaches, although characterized by sub-optimal spatial resolution relative to fMRI, can capture brain function during naturalistic design tasks (as will be discussed further below), and have been an emerging recent tool in design neurocognition studies. With findings interpreted in the context of design theory, these techniques hold promise for a multi-level understanding of how neurocognitive systems enable design thinking, which—in turn—opens new avenues toward its improvement. We provide a succinct summary of some of the research in the cognitive neuroscience of design that has employed these techniques next.

## 3. Using neuroscience techniques to understand design thinking

Among the first studies to investigate the neural bases of design cognition was an examination of the potential differences in brain engagement between designing and problem solving [12] using fMRI. Participants with varying design experience were presented with a number of design and problem solving tasks in counterbalanced order and were asked to first study and then solve each task using a trackball mouse while undergoing fMRI. The results revealed distinct patterns of activity in prefrontal cortex between the two types of cognitive tasks. Additional fMRI studies have examined neural variability during conceptual design problem solving in the presence or absence of inspirational stimuli. In one example, participants performed a concept generation task while either presented with stimuli that were more or less related to the problem space to use as inspiration or without the presence of such stimuli [13]. The results of that study revealed that inspirational stimuli promoted idea generation, while eliciting distinct patterns of brain activation during problem solving compared to trials without inspirational stimuli. Neural differences between generating ideas and evaluating them have also been reported during a graphic design task, where participants were asked to alternative between designing comic book covers and evaluating their designs [14]. Lastly, fMRI has been used as a tool to evaluate the impact of built environments on the brain. For example, dissociable anterior-posterior neural patterns have been reported while architects are evaluating contemplative (relative to functional) spaces in architectural design [15]. Overall, this research has revealed that designing appears to differ at the neural level from other cognitive processes such as problem solving or various aspects of creative thinking (e.g., idea generation or evaluation), and it can preferentially elicit prefrontal cortical responses depending on the design task. Although these early studies have been impactful in describing the possible candidate mechanisms of design at the neural level, the localizationist approach favored in this past work has limited our understanding of how these neural systems interact and how these processes take place during real-life design tasks. That is, this work has identified the selective contributions of some brain regions to design thinking relative to other regions, but has not yet examined how these areas interact dynamically in the process of designing.

These early functional neuroimaging approaches are additionally challenged by the technique's limited temporal resolution, as they fail to capture one of the most critical aspects of designing, namely, its inherently multifaceted and rapidly evolving temporal nature. The focus on the temporal variability of neural processing during design has been the target of investigations within design neurocognition that have employed EEG or ERP recordings. For example, recent experimental work has shown that higher alpha-band activity over temporal and occipital regions can distinguish between open-ended problem descriptions, relative to close-ended and decision-focused problem descriptions during design problem-solving in expert designers [16]. Similarly, EEG components have been shown to be reliable indicators for the measurement of effort, fatigue, and concentration, as evaluated while participants performed conceptual design tasks on a sketchpad under EEG monitoring [17]. EEG paradigms have also focused on cognitive processes during design more directly. For instance, expert designer's visual attention and associative processes during design have shown that more posterior occipitoparietal and dorsal central regions were engaged during visual association relative to visual attention tasks [18]. Lastly, EEG signals have been used to differentiate between different groups of designers with different types of expertise across problem-solving and design tasks—with mechanical engineers showing different patterns of local activity and temporal distribution of that activity across prefrontal and occipitotemporal regions relative to industrial designers [9]. This work has shown that EEG patterns can distinguish among different cognitive processes in design, corroborating behavioral evidence and propositions put forth by design theory. On the other hand, these approaches are limited by the poor spatial resolution of the technique and the challenges of pairing behavioral measurements of the design process (e.g., through verbal protocols) with neurophysiological measures due to motion-induced artifacts—although ongoing methodological advances hold potential for addressing these issues in future research (see [9]).

To address the high-costs and potential limitations of functional brain imaging and EEG for design studies in ecologically valid, real-world settings, recent research in design neurocognition has also used fNIRS as a method of capturing functional brain changes during design in real-time. A benefit of fNIRS paradigms is that the participant can freely move, speak, interact with others, and use devices during tasks, thus, allowing for data collection in naturalistic educational and professional settings. Thus far, only a handful of pilot studies have used this approach to examine design thinking, though the preliminary results suggest that fNIRS measurements can detect cortical shifts as a result of design constraints [19], as well as differentiate between groups of design experts using brainstorming, morphological analysis, or Theory-of-Inventive-Problem-Solving (TRIZ) strategies—a method of idea generation that entails finding the underlying principles supporting past solutions to a similar problems and utilizing them in the current problem circumstances [20].

Though not exhaustive, the above review of the evidence from the emerging field of design neurocognition has generated interesting preliminary findings regarding the neural bases of design thinking that promise to differentiate among competing theoretical propositions on the elements of the design process, thus impacting design theory, education, and practice (for a recent comprehensive review of other neurophysiological studies of design cognition beyond brain-based measures, see [21]). This emerging field of research, in turn, presents an interesting challenge for experimental cognitive neuroscience, by contextualizing and probing established findings about brain function within an applied field of inquiry. On the other hand, design neurocognition approaches have remained largely descriptive and have not been explicitly linked to established neurocognitive mechanisms underlying complex higher-order thinking as founded on rigorous experimental paradigms. As a result, a systematic framework for understanding design cognition backed by neuroscience findings is lacking. We detail these challenges for the field, as well as the opportunities they bring forth, in the next section.

## 4. Challenges and opportunities for the cognitive neuroscience of design

Although designers' behaviors are well-described within the design literature, due to its complexity, comprehensive examinations of design thinking through cognitive neuroscience methods have lagged behind. One of the key challenges of research in design neurocognition as detailed above is that it has largely embraced a piecemeal, reductionist approach where aspects of design cognition (e.g., problem understanding, idea generation, memory and decision making) are examined in isolation by means of paradigms loosely based on experimental cognitive psychology and neuroscience methods [8],[13]. However, designing is a real-world, complex system of interacting activities that occur over time; thus, designing cannot be decomposed to subsystems without losing its fundamental characteristics [22]. Indeed, our understanding of the neurocognitive processes underlying design thinking is profoundly incomplete as it has—with few exceptions [9],[14]—mainly relied on decontextualized and fragmented laboratory tasks that ostensibly serve as models of real-world cognitive processing. A significant disadvantage of these methodologies is that they fail to capture comprehensively how humans creatively problem-solve in their everyday lives—how they design new products and services that shape and propel our world. Moreover, the selective application of cognitive neuroscience technologies for the collection and reporting of descriptive (and, at times, individual participant) data without the adoption of proper statistical and power analyses methods, has limited the rigor and reproducibility of the reported findings.

We invite the field to address these shortcomings through meaningful collaborations between cognitive neuroscience and design neurocognition researchers. We propose that advancing our understanding of design thinking can only be achieved through *in vivo* examinations of the neurocognitive processes taking place while designers work on real-world design problems. Toward this goal, one potential avenue for future research entails the combination of verbal protocol analysis and multi-modal neuroimaging-based methods, such as fMRI, with concurrent EEG or other psychophysiological measures (e.g., eye-tracking, skin conductance). Recent technological advances have made it possible for participants to complete real-world design tasks using MRI-compatible tablets for sketching and design problem-solving tasks [14]; similar tools have been used to investigate musical improvisation [23]. This progress has allowed for the collection of reliable verbal responses under fMRI while maintaining superior image quality [24]–[28]. Additionally, with network neuroscience approaches to the analysis of brain imaging data taking the lead within cognitive neuroscience for the study of thought and behavior relative to traditional localizationist views, there is strong potential for the evaluation of complex processes like design at the systems level. If such measures are paired with real-world design tasks, the emerging findings can support a neurocognitive framework toward understanding design thinking that focuses on how the dynamic process of designing takes place within the human cognitive architecture. In turn, such findings can offer perspective on how well-established processes from experimental laboratory cognitive neuroscience research apply to the underpinnings of higher-order cognition in real-world tasks.

Moreover, a clear and methodologically-rigorous account of the neural systems involved in design cognition creates new opportunities for determining whether we can actively augment and improve design thinking through altering brain function. For example, task-synchronous manipulations of designers' brain activity can be achieved through behavioral, neurofeedback, or non-invasive brain stimulation interventions, such as transcranial direct current stimulation (tDCS) and transcranial alternating current stimulation (tACS). By combining the complementary fields of cognitive neuroscience and design, while leveraging recent technological and methodological advances, research on the cognitive neuroscience of design can generate—using real-world design problems—an unprecedented dataset of neurocognitive measures that can provide a unique understanding of the complex cognitive and brain systems enabling design thinking. The significance of this new knowledge can, then, be manifested in the opportunity to develop and implement new techniques toward cultivating and improving design thinking.

## 5. Conclusions

Designing is a real-world, dynamic system of interacting, temporally-distributed activities and is among the most complex aspects of higher-order human cognition. In this opinion paper we propose that cognitive neuroscience studies of design need to embrace this complexity and examine the attendant neurocognitive processes taking place while designers work on real-world design problems. Such work can establish a neurocognitive framework toward understanding design thinking that focuses on how the dynamic process of designing takes place within the human cognitive architecture. This emerging knowledge can support the development of new techniques to augment real-world design thinking by causally altering the neural networks involved in design. The productive pairing of cognitive neuroscience and design neurocognition approaches will pave the way toward developing new techniques for improving design thinking, which can be applied to individual designers or can be extended to design teams in future work.
